# A Variable CD3^+^ T-Cell Frequency in Peripheral Blood Lymphocytes Associated with Type 1 Diabetes Mellitus Development in the LEW.1AR1-*iddm* Rat

**DOI:** 10.1371/journal.pone.0064305

**Published:** 2013-05-22

**Authors:** Tanja Arndt, Anne Jörns, Heike Weiss, Markus Tiedge, Hans-Jürgen Hedrich, Sigurd Lenzen, Dirk Wedekind

**Affiliations:** 1 Institute of Clinical Biochemistry, Hannover Medical School, Hannover, Germany; 2 Institute for Laboratory Animal Science, Hannover Medical School, Hannover, Germany; 3 Centre of Anatomy, Hannover Medical School, Hannover, Germany; 4 Institute of Medical Biochemistry and Molecular Biology, University of Rostock, Rostock, Germany; University of Bremen, Germany

## Abstract

**Purpose:**

The LEW.1AR1-*iddm* rat is an animal model of human type 1 diabetes mellitus (T1DM), which arose through a spontaneous mutation within the MHC-congenic inbred strain LEW.1AR1 (*RT1^r2^*). In contrast to the diabetes-resistant LEW.1AR1 background strain in LEW.1AR1-*iddm* rats a highly variable T-cell frequency could be observed in peripheral blood lymphocytes (PBLs).

**Methods:**

In this study we therefore characterised the T-cell repertoire within the PBLs of the two strains by flow cytometry analysis and identified the CD3^+^ T-cell phenotype and its possible linkage to diabetes susceptibility. To map loci conferring susceptibility to variable CD3^+^ T-cell frequency, backcross strains (N2) were generated with the genetically divergent BN and PAR rats for microsatellite analysis.

**Results:**

The LEW.1AR1-*iddm* rat strain was characterised by a higher variability of CD3^+^ T-cells in PBLs along with a slightly decreased mean value compared to the LEW.1AR1 background strain. The reason for this reduction was a decrease in the CD4^+^ T-cell count while the CD8^+^ T-cell proportion remained unchanged. However, both T-cell subpopulations showed a high variability. This resulted in a lower CD4^+^/CD8^+^ T-cell ratio than in LEW.1AR1 rats. Like LEW.1AR1-*iddm* rats all animals of the backcross populations, N2 BN and N2 PAR rats, also showed large variations of the CD3^+^ T-cell frequency. The phenotype of variable CD3^+^ T-cell frequency mapped to the telomeric region of chromosome 1 (RNO1), which is identical with the already known *Iddm8* diabetes susceptibility region. The data indicate that a variable CD3^+^ T-cell frequency in PBLs is genetically linked to diabetes susceptibility in the LEW.1AR1-*iddm* rat.

**Conclusion:**

The T-cell variability in PBLs could be related to the previously reported imbalance between regulatory and effector T-cell populations which results in beta-cell autoimmunity. Since similar T-cell phenotypes have also been described in human T1DM the identification of the functional role of the observed variable CD3^+^ T-cell frequency may help to understand the mechanisms of autoimmunity in T1DM.

## Introduction

Type 1 diabetes mellitus (T1DM) is a multifactorial disease in which a predisposing genetic background as well as environmental factors ultimately lead to an autoimmune destruction of the pancreatic beta cells [1]. Animal models play an important role for the understanding of the pathogenesis of T1DM because they permit combining genetic and functional characterisation of the syndrome [2]. The LEW.1AR1-*iddm* rat is a model for human T1DM, which arose through a spontaneous mutation in the intra-MHC recombinant inbred strain LEW.1AR1 (*RT1^r2^*, *RT1-A^a^, RT1-B/D^u^, RT1-C^u^*) [3]. This model shows an apoptotic beta-cell destruction, induced by proinflammatory cytokines released from islet infiltrating immune cells [4]. The autoimmune nature of the diabetic syndrome has been proven by adoptive transfer experiments [5], [6].

The diabetic syndrome of the LEW.1AR1-*iddm* rat follows an autosomal recessive mode of inheritance with an incomplete penetrance of the mutant phenotype of about 60% [3], [4]. Three T1DM susceptibility loci in the LEW.1AR1-*iddm* model have been discovered by genome wide linkage analysis using a [(BN×LEW.1AR1-*iddm*) ×LEW.1AR1-*iddm*] N2 (N2 BN) population [7]. One locus mapped to RNO20p12 within the MHC (Major Histocompatibility Complex) region which provides T1DM susceptibility also in humans (*IDDM1*), the NOD mouse (*Idd1*), the BB rat and the KDP rat models (*Iddm1*) [8]. Thus, the MHC haplotype plays a pivotal role in permitting T1DM development [9].

In the LEW.1AR1-*iddm* rat two further *Iddm* loci reside on RNO1. The *Iddm8* locus was discovered within RNO1q51–55 at the telomeric end and *Iddm9* could be localized in RNO1p11–1q11 near the centromer using the N2 BN backcross population. In an additional [(PAR×LEW.1AR1-*iddm*)×LEW.1AR1-*iddm*] N2 (N2 PAR) population the *Iddm1* and *Iddm8* loci could be confirmed in this genetically divergent strain [10].

The LEW.1AR1-*iddm* rats show mean values of CD3^+^ T-cells in peripheral blood around 50% by flow cytometric analysis [3]. In the present study the detailed analysis of immune cells in peripheral blood indicated that LEW.1AR1-*iddm* rats compared to the diabetes-resistant LEW.1AR1 background strain showed a slight decrease of the mean value of around 10% (non-diabetic LEW.1AR1-*iddm* rats) and 20% (diabetic LEW1AR1-*iddm* rats) and a more variable CD3^+^ T-cell frequency than the background strain. The phenotype of the ‘variable CD3^+^ T-cell frequency’ was characterized and the responsible locus for this trait could be mapped within *Iddm8* in two N2 cohorts generated with the genetically divergent BN and PAR strains. Our data provide evidence that the mutation within the *Iddm8* region on RNO1 not only confers susceptibility to T1DM but also to the variable CD3^+^ T-cell frequency in blood.

## Materials and Methods

### Animals

All rats were housed under specific pathogen free (SPF) conditions in the same hygiene unit at the Central Animal Facility of Hannover Medical School (Ztm). They were regularly monitored for infection by typical viral pathogens and were shown to be serologically negative for Hanta, Kilham rat, PVM, Reo3, Sendai, SDA, rat corona, Theiler’s encephalomyelitis, and Toolan’s (H1) viruses [3], [11]. The rats were held in groups of three animals under a 14∶10 light-dark cycle, 55±5% humidity, in type IV Makrolon cages (Techniplast, Hohenpeißenberg, Germany) on a standard softwood bedding (Altromin ¾), with free access to sterilised standard laboratory chow (diet No. 1324, Altromin, Lippe, Germany) and water.

The following strains were analysed by flow cytometry: LEW.1AR1-*iddm* (n = 34 diabetic; n = 32 non-diabetic), LEW.1AR1 (n = 12), BN (n = 10) and PAR (n = 8) and all generated crosses as described (F1 BN: n = 6; N2 BN: n = 155; F1 PAR: n = 12; N2 PAR: n = 151). Blood was taken from all animals at an age between 35–110 days (from LEW.1AR1-*iddm* rats and N2 rats after diabetes onset, from non-diabetic LEW.1AR1-*iddm*, F1 and N2 rats between day 90 and 110 and from the background strain LEW.1AR1 between day 35 and 110). BN and PAR backcross populations were generated as described below. F1 animals were generated by mating diabetic male LEW.1AR1-*iddm* rats with female BN (*RT1^n^*) and PAR (*RT1^g^*) rats to analyse the LEW.1AR1-*iddm* rat for susceptibility loci for the variable CD3^+^ T-cell frequency. Notably, none of the BN rats or PAR rats developed diabetes. The female offspring of the intercrosses (LEW.1AR1-*iddm*×BN) F1 and (LEW.1AR1-*iddm*×PAR) F1 was backcrossed to diabetic male LEW.1AR1-*iddm* rats. N2 BN and N2 PAR animals were genotyped by microsatellite analysis. Blood glucose concentrations were checked twice weekly until day 120 of life (Glucometer Elite™, Bayer, Leverkusen, Germany). Diabetic animals were sacrificed within 48 h after onset of hyperglycemia (≥ 10 mmol/l) for preparation of genomic DNA from tail, ear, spleen, and thymus. The same procedure was applied to non-diabetic animals at the age of 120 days.

Experimental procedures were performed according to the German Animal Welfare Act (*Tierschutzgesetz*, § 4) and approved by the Local Institutional Animal Care and Research Advisory Committee of Hannover Medical School and the Lower Saxony State Office for Consumer Protection and Food Safety (Approval ID: 42500/1H).

### Pancreas Morphology

All pancreases of the diabetic and non-diabetic animals of the different strains and cross populations were analysed morphologically for immune cell infiltration. Independent from the changes in the CD3^+^ T-cell frequency in PBLs (peripheral blood lymphocytes) the pancreatic islets of all diabetic animals were severely infiltrated by T-cells and macrophages as main immune cell types. The pancreatic islets from the non-diabetic animals remained unaffected.

### DNA Preparation

Genomic DNA was extracted from the tissues using the NucleoSpin™ Tissue kit (Macherey-Nagel, Düren, Germany) according to the manufacturer’s instructions.

### Flow Cytometry

In order to determine the different lymphocyte subpopulations in peripheral blood flow cytometric analyses were performed using the following labelled monoclonal antibodies: CD3 (G4.18) PE, CD4 (OX-38) FITC, (Becton Dickinson, Heidelberg, Germany); CD8 (OX-8) FITC, CD8 (OX-8) PE, (Serotec, Düsseldorf, Germany). 50 µl blood were prepared for immune cell analysis by 2–3×lysis in 2 ml lysis buffer (160 mmol NH_4_, 0.1 mmol EDTA, 12 mmol NaHCO_3_) and centrifuged (200×g) for 4 min each. Thereafter, the cell pellet was washed twice with FACS buffer (phosphate buffered saline, 0.03% sodium azide, 0.1% bovine serum albumin) and centrifuged again. The pellet was resuspended in 20 µl diluted antibody solution and incubated for 30 min at 4°C. After washing the cells twice with 2 ml FACS buffer the cell pellet was resuspended in 200 µl FACS buffer and measured in a flow cytometer (Becton Dickinson). Cell characteristics (size and granularity) and antibody staining of living cells were assessed. Data processing was carried out using the Cellquest 3.0.1 Software. A gate for lymphocytes was created based on size and granularity of peripheral blood cells.

### Microsatellite Analyses

All oligonucleotide primers used in this study were developed and used as described previously for genome wide mapping of the phenotype ‘diabetes’ [7], [10]. The PCR reaction was performed according to manufacturer’s instructions (Peqlab Biotechnologie, Erlangen, Germany) with 100 µg DNA template per well in a 96-well plate (MultiRigid Ultra Plates™, Roth, Karlsruhe, Germany) using a PTC-200 thermocycler (Biozym, Hess. Oldendorf, Germany). The PCR conditions were 4 min at 94°C; 35 cycles: 15 s at 94°C, 1 min at 55°C with the exception of *D1Ztm1* (57°C) and *D1Ztm2* (53.2°C), 2 min at 72°C; 7 min final extension at 72°C. PCR products were analysed by electrophoresis in 3% NuSieve™ agarose gels (Biozym, Hess. Oldendorf, Germany). Gels were stained using Gelstar™ (Cambrex, Apen, Germany) and documented by UV light illumination at 312 nm.

### Statistical Analyses

Linkage analysis was performed using the JoinMap V 2.0 program (Agricultural Research Dept., Wageningen, Netherlands). The LOD (logarithm of the odds) scores of the susceptibility regions for the variable T-cell frequency were calculated using the R/qtl program kindly provided by Dr. K. Browman (Dept. of Biostatistics, Johns Hopkins University, Baltimore, MD) as used before for identification of the mutation in other disease models [12]–[15]. E/M algorithms estimated susceptibility regions in a binary model using the CD3^+^ T-cell frequency (T-cell frequency >70% and <40%) of the animals as a trait. A permutation test was performed to calculate the threshold value for significance (LOD score >2.3) [16]. Statistical analysis of flow cytometry (mean values, ANOVA plus Tukey’s post test and coefficient of variation (CV)) was calculated using GraphPad Prism 5 software.

## Results

### Frequency of T-cells in PBLs in the LEW.1AR1-*iddm* (Diabetic and Non-diabetic) Rats and the Background Strain LEW.1AR1

**Figure 1:**
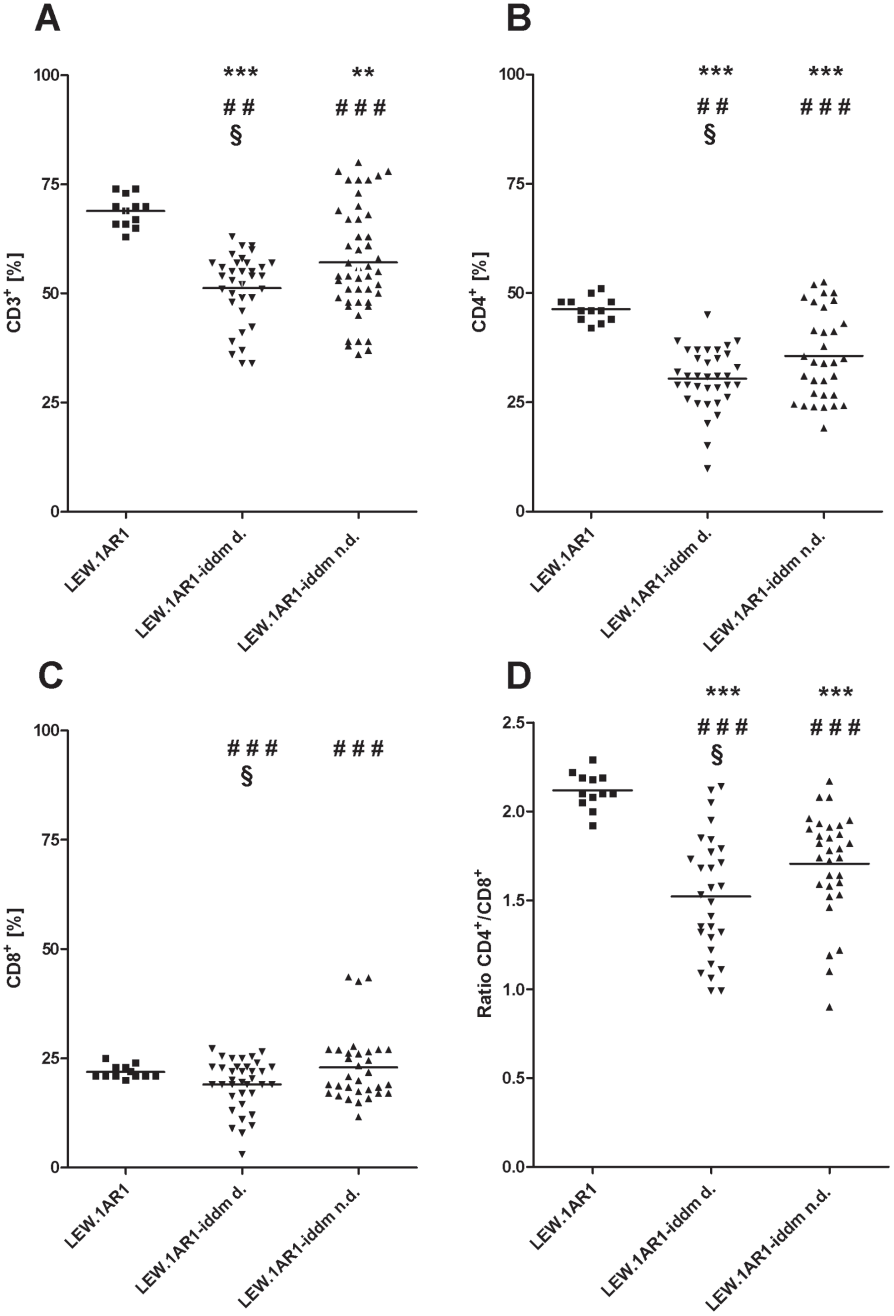
The percentages of T-cell subpopulations within PBLs of LEW.1AR1 and LEW.1AR1-*iddm* rats (d. = diabetic animals; n.d. = non-diabetic animals). The percentages of (A) CD3^+^, (B) CD4^+^, (C) CD8^+^ T-cells, and (D) CD4^+^/CD8^+^ T-cell ratio were determined in PBLs by flow cytometric analysis from rats at an age between 35 and 110 days. Each symbol represents the T-cell value for a single animal. The number of animals was for LEW.1AR1 (n = 12), LEW.1AR1-*iddm* diabetic (n = 34) and LEW.1AR1-*iddm* non-diabetic (n = 32). The mean values for the different groups are represented by the horizontal bar in the graphs and were compared statistically. **p<0.01/***p<0.0001 LEW.1AR1 compared to LEW.1AR1-*iddm*, ^§^p<0.05 diabetic compared to non-diabetic LEW.1AR1-*iddm* rats, and ^##^p<0.01/^###^p<0.0001 for range of percentage LEW.1AR1 group compared to LEW.1AR1-*iddm* group, (ANOVA plus Tukey’s post test).

The percentage of CD3^+^ T-cells in PBLs within the LEW.1AR1 background population varied between 63% and 74% (mean ± SEM: 69±1%, CV (coefficient of variation): 5.3) (Fig. 1A). In contrast, the variability of the T-cell frequency in PBLs within the diabetic and non-diabetic LEW.1AR1-*iddm* population was higher than in the LEW.1AR1 strain. Significant differences of the average percentage could be detected not only between the inbred strains LEW.1AR1 and LEW.1AR1-*iddm*, but also between the diabetic and the non-diabetic LEW.1AR1-*iddm* group (Fig. 1A). The percentage of CD3^+^ T-cells in PBLs within the diabetic LEW.1AR1-*iddm* group was in the range of 34% to 63% (51±1%, p<0.001, CV: 15.8), while within the non-diabetic LEW.1AR1-*iddm* group this value varied between 36% and 80% (59±2%, p<0.02, CV: 22.9). Additionally, a significant decrease of the CD3^+^ T-cells could be observed in the diabetic LEW.1AR1-*iddm* group as compared to the non-diabetic LEW.1AR1-*iddm* group (p<0.001), but the variability in both groups was comparable.

In LEW.1AR1 rats the percentage of CD4^+^ T-cells in PBLs varied within a narrow range between 42% and 51% (46±1%, CV: 6.0) (Fig. 1B). In contrast, diabetic and non-diabetic LEW.1AR1-*iddm* rats exhibited not only a higher variability but also a lower percentage of CD4^+^ T-cells in PBLs. Notably, these differences were also observed between the diabetic and the non-diabetic LEW.1AR1-*iddm* rats (Fig. 1B). The percentage of CD4^+^ T-cells in PBLs in the diabetic LEW.1AR1-*iddm* group was in the range of 10% to 45% (30±1%, p<0.001, CV: 23.0), while the frequency of CD4^+^ T-cells in PBLs within the non-diabetic LEW.1AR1-*iddm* group varied between 19% and 52% (36±2%, p<0.001, CV: 28.3). A significant decrease of CD4^+^ T-cells could be also observed between the diabetic LEW.1AR1-*iddm* cohort and the non-diabetic LEW.1AR1-*iddm* cohort (p<0.02) with comparable variability.

The percentage of CD8^+^ T-cells in PBLs within the LEW.1AR1 control cohort showed a low variability between 20% and 25% (22±0%, CV: 6.9) (Fig. 1C). In contrast, the variability of the T-cell frequency in PBLs within the diabetic and non-diabetic LEW.1AR1-*iddm* population was very high but significant differences of the mean percentage could not be detected between LEW.1AR1 and both diabetic and non-diabetic LEW.1AR1-*iddm* rats (Fig. 1C). The percentage of CD8^+^ T-cells in PBLs within the diabetic LEW.1AR1-*iddm* group was in a range between 3% and 27% (19±1%, CV: 30.4), while the frequency of CD8^+^ T-cells in PBLs within the non-diabetic LEW.1AR1-*iddm* group varied between 12% and 44% (23±1%, CV: 34.8). A significant decrease of CD8^+^ T-cells could only be observed in the diabetic LEW.1AR1-*iddm* group compared to the non-diabetic LEW.1AR1-*iddm* group (p<0.02) with similar variability in both groups.

### Ratio of CD4^+^/CD8^+^ T-cells in PBLs of LEW.1AR1 and LEW.1AR1-*iddm* Rats

Because of the variability in each single animal it was necessary to calculate the CD4^+^/CD8^+^ T-cell ratio for each animal. From these a mean value was calculated for the CD4^+^/CD8^+^ T-cell ratio of each animal group.

The CD4^+^/CD8^+^ T-cell ratio in PBLs of diabetes resistant LEW.1AR1 rats was on average 2.1±0.1 (Fig. 1D). The diabetic LEW.1AR1-*iddm* group had a significantly lower CD4^+^/CD8^+^ T-cell ratio within PBLs (1.5±0.1, p<0.001) with a higher variability (1.0 to 2.1, CV: 22.4) compared to the LEW.1AR1 background strain (2.1±0.3, CV: 4.8) (Fig. 1D). The non-diabetic LEW.1AR1-*iddm* rats also showed a significantly lower CD4^+^/CD8^+^ T-cell ratio (1.7±0.1, p<0.001) with a higher variability (0.9 to 2.2, CV: 17.3) compared to the background strain. But there was no difference in the variability of the CD4^+^/CD8^+^ T-cell ratio in PBLs between diabetic and non-diabetic LEW.1AR1-*iddm* rats. Interestingly, the CD4^+^/CD8^+^ T-cell ratio was significantly higher in non-diabetic LEW.1AR1-*iddm* rats (1.7±0.1, p<0.05) than in diabetic LEW.1AR1-*iddm* rats (1.5±0.1).

### Distribution and Inheritance of the CD3^+^ T-cell Frequency in PBLs within Different Rat Inbred Strains, Intercrosses (F1) and Backcrosses (N2)

**Figure 2:**
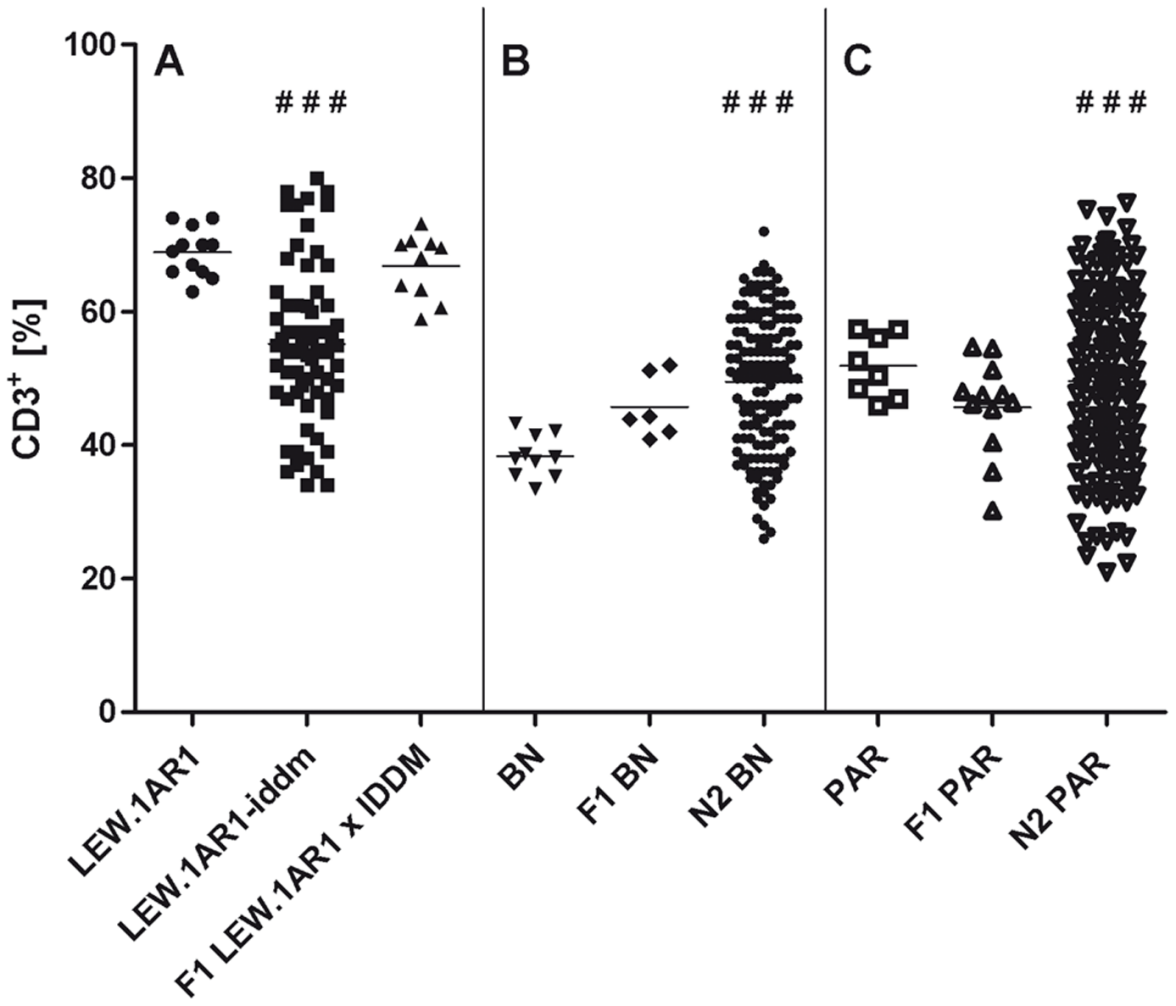
The percentages of CD3^+^ T-cells within PBLs of different rat strains and crosses. CD3^+^ T-cells were measured in PBLs from (A) LEW.1AR1 and LEW.1AR1-*iddm* and the F1 populations as well as the backcross population (N2) with the (B) BN and (C) PAR strain by flow cytometric analysis at the age between 35–110 days. Each symbol represents the T-cell value for a single animal. The number of animals was for LEW.1AR1 (n = 12), LEW.1AR1-*iddm* (n = 66), F1 (LEW.1AR1×LEW.1AR1-*iddm*) (n = 10), BN (n = 10), F1 (BN×LEW.1AR1-*iddm*) (n = 6), N2 BN (n = 155), PAR (n = 8), F1 (PAR×LEW.1AR1-*iddm*) (n = 12), and N2 PAR (n = 151). The mean values for the different groups are represented by the horizontal bar in the graphs. **^###^**p<0.0001 range of percentage (LEW.1AR1 group compared to LEW.1AR1-*iddm* group, BN group compared to N2 BN group and PAR group compared to N2 PAR group; ANOVA plus Tukey’s post test).

As described before, the variance of the CD3^+^ T-cell frequency in PBLs within the cohort of the mutated LEW.1AR1-*iddm* rats (CV: 21.4) was significantly higher than that of the LEW.1AR1 group (CV: 5.2, p<0.01, Fig. 2A). The mean of the CD3^+^ T-cell frequency in the F1 animals of a (LEW.1AR1-*iddm*×LEW.1AR1) cross as well as the variability (69±2%, CV: 7.2) did not differ from the background strain LEW.1AR1, indicating an autosomal recessive mode of inheritance.

Although the inbred strains LEW.1AR1, BN, and PAR differed in their average CD3^+^ T-cell frequency within PBLs the variability of the CD3^+^ T-cell frequency was comparatively low in these three strains (LEW.1AR1∶69±1%, CV: 5.2; BN: 38±1%, CV: 8.3; PAR: 52±2%, CV: 9.0) (p≥0.5). Also the intercrosses (BN×LEW.1AR1-*iddm*) F1 and (PAR×LEW.1AR1-*iddm*) F1 did not differ significantly from BN and PAR strains, respectively in the variability of the CD3^+^ T-cell frequency (p≥0.5). The frequency of CD3^+^ T-cells in PBLs in the (BN×LEW.1AR1-*iddm*) F1 progeny was 46±3% (CV: 12.4) (Fig. 2B) and in the (PAR×LEW.1AR1-*iddm*) F1 progeny 46±2% (CV: 11.7) (Fig. 2C). The frequency and the variance in both F1 cohorts did not differ significantly from the BN and PAR rats but it did from the LEW.1AR1-*iddm* rats, indicating a recessive mode of inheritance again.

For mapping the causative mutation a [(BN×LEW.1AR1-*iddm*) ×LEW.1AR1-*iddm*] N2 (N2 BN) population and a [(PAR×LEW.1AR1-*iddm*) ×LEW.1AR1-*iddm*] N2 (N2 PAR) population were generated. According to the Mendelian rules it could be expected that 50% of the N2 population display a change in the T-cell frequency (mean values, variability) in the PBLs. In fact, the percentage of T-cells varied between 26 and 72% with a mean of 44±2% (CV: 20.2) in the N2 BN population and between 21 and 76% with a mean of 40±1% (CV: 25.96%) in the N2 PAR population (Fig. 2B and Fig. 2C). In both cases the CV differed significantly (p<0.03) from the F1 population and was identical to that of the LEW.1AR1-*iddm* rats.

### Association of Microsatellite Markers with Diabetes Manifestation and CD3^+^ T-cell Frequency in PBLs

We mapped the phenotype of the variable CD3^+^ T-cell frequency in the LEW.1AR1-*iddm* strain using 157 informative markers with an intermarker distance of about 20 cM over the whole genome [7], [10]. In the region of interest on RNO1 a denser mapping was performed using microsatellite markers with an intermarker distance of about 1 cM.

**Figure 3:**
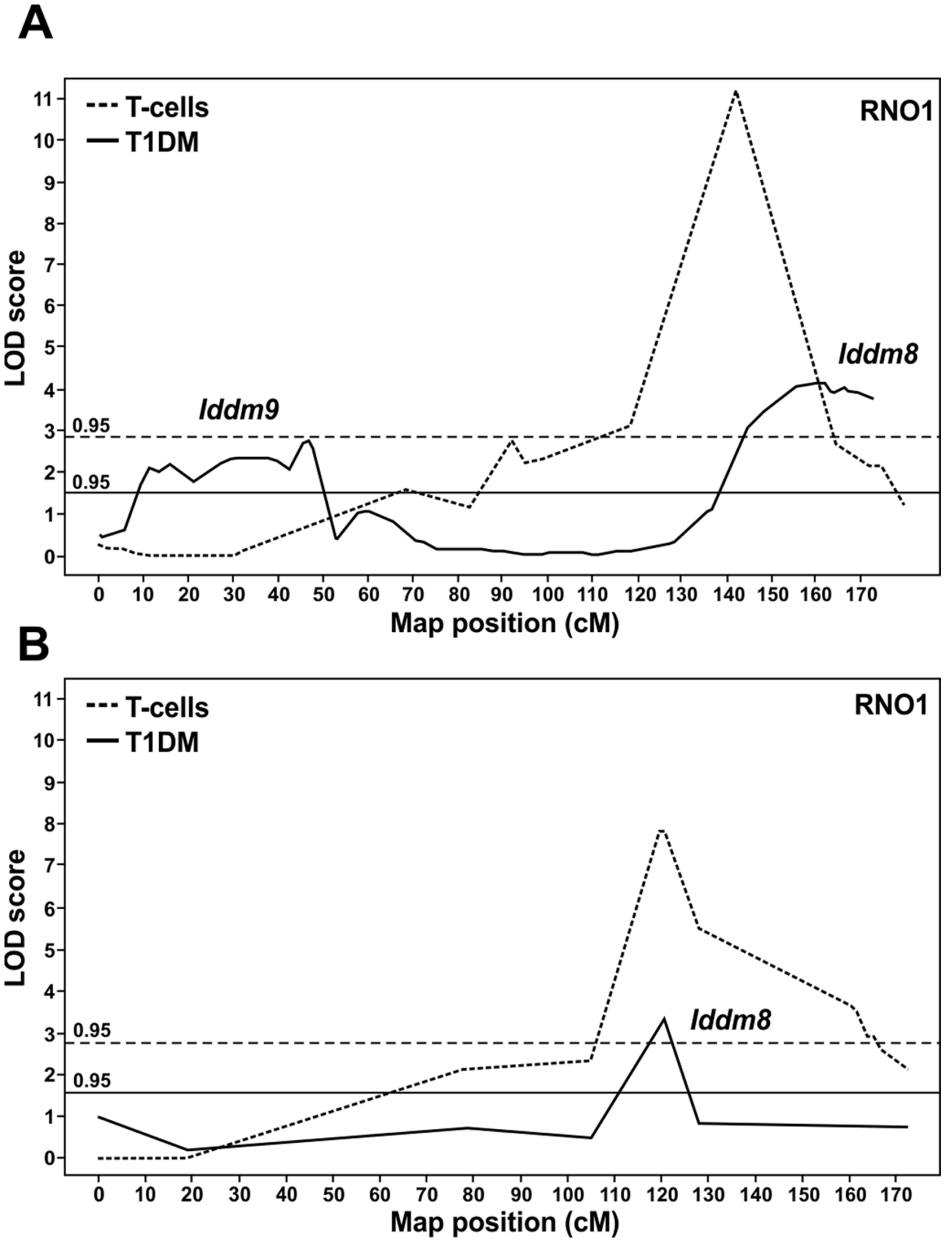
Association of microsatellite markers on RNO1 with the diabetes syndrome and T-cell frequency within PBLs. The LOD scores were estimated using E/M algorithms. (A) Analyses of the [(BN×LEW.1AR1-*iddm*) ×LEW.1AR1-*iddm*] N2 showed a peak marker *D1Ztm1* with a LOD score of 4.13 for diabetes (solid line), which was located at 162.1 cM/247.3 Mb. The variability of the T-cell frequency (dotted line) was mapped to the telomeric end of RNO1 (135 to 145 cM). The mapped locus is closely linked to *Iddm8*. (B) Analyses of the [(PAR×LEW.1AR1-*iddm*) ×LEW.1AR1-*iddm*] N2 showed a peak marker *D1Ztm17* with a LOD score of 3.9 for diabetes and is located at 120.9 cM/213.13 Mb (solid line). The variability of the T-cells frequency (dotted line) was mapped to the telomeric end of chromosome 1 (125 to 135 cM). The mapped locus was closely linked to *Iddm8*. A permutation test considered a LOD score of >1.5 as significant for association with diabetes (solid line) and a LOD score of >2.8 as significant for association with T-cell frequency (dotted line).

Linkage analysis of the trait ‘variable CD3^+^ T-cell frequency’ in a N2 BN cohort identified one susceptibility locus on RNO1 (Fig. 3A, dotted line), while for the trait ‘T1DM’ two susceptibility loci could be mapped on RNO1, *Iddm8* and *Iddm9* (Fig. 3A, solid line) [7]. The peak of this locus of the variable CD3^+^ T-cell frequency was located within the already identified *Iddm8* region at the telomeric end of RNO1 at about 145 cM (Fig. 3A, dotted line). The linkage analysis of an N2 PAR population showed only one T1DM susceptibility locus in the rat, overlapping with *Iddm8* (Fig. 3B, solid line) [10]. In this N2 PAR population the peak of the locus of the trait ‘variable CD3^+^ T-cell frequency’ could also be mapped within the *Iddm8* region on the telomeric end of RNO1 at about 120 cM (Fig. 3B, dotted line). Thus there is a clear overlap between the susceptibility loci for T1DM manifestation and variable CD3^+^ T-cell frequency.

## Discussion

The present study was prompted by the observation that the LEW.1AR1-*iddm* strain showed a high variability of the T-cells in the PBLs with a significantly lower average of the CD3^+^ T-cell proportion. This raised the questions (1) whether the trait ‘variable CD3^+^ T-cell frequency’ is specific for the diabetes susceptible LEW.1AR1-*iddm* strain and (2) whether this trait could be mapped to a genomic region that is linked to diabetes susceptibility.

### The Phenotype ‘variable CD3^+^ T-cell Frequency’ is Specific for the LEW.1AR1-*iddm* Rat Strain

LEW.1AR1-*iddm* rats showed a significant reduction in the average CD3^+^ T-cell frequency compared to the background strain with a great variability ranging from 34% to 80%. Moreover, the CD3^+^ T-cell frequency was also significantly reduced in diabetic LEW.1AR1-*iddm* rats compared to the non-diabetic ones.

It is generally accepted that a decrease of the peripheral T-cell repertoire with a particular emphasis on the CD4^+^/CD8^+^ T-cell ratio affects the balance between regulatory and effector cells and thus triggers mechanisms of autoimmunity. In the BB rat model, a pronounced reduction of all peripheral T-cells has been reported to accompany diabetes manifestation [17]–[21]. In the KDP rat a mutation of the *Cblb* gene has been described, which should affect T-cell activation but a verification of the functional relevance of this mutation has not been provided so far [22]. Interesting is also the observation that CD3^+^ and CD4^+^ T-cells as well as the CD4^+^/CD8^+^ ratio were significantly lower in diabetic dogs [23].

In humans results of several studies indicate changes of the distribution of peripheral T-cells at diagnosis and during on-going autoimmunity when compared to healthy controls [24], [25]. Interestingly, the variability of the CD3^+^ T-cells was similar to that in the LEW.1AR1-*iddm* rat [26]–[29]. However, there is still controversy about the T-cell subpopulation(s) that are increased or decreased, a phenomenon that could be summarized under the term ‘variability’ [30]–[33]. In conclusion, there is consensus that changes in the T-cell distribution contribute to T1DM manifestation also in humans.

### Influence of Changes in T-cell Frequency on the CD4^+^/CD8^+^ Ratio

Changes in the T-cell frequency of the LEW.1AR1-*iddm* population had a direct effect on the CD4^+^/CD8^+^ ratio. A reduced ratio of CD4^+^/CD8^+^ peripheral T-cells found in diabetic and non-diabetic LEW.1AR1-*iddm* rats was mainly due to a decrease of the CD4^+^ T-cell number whereas the CD8^+^ T-cell number remained stable. Nevertheless, both CD4^+^ and CD8^+^ T-cell numbers were highly variable. Here again the variability of the CD4^+^/CD8^+^ ratio within the LEW.1AR1-*iddm* cohort was much higher than in the background strain. Furthermore, differences between non-diabetic and diabetic animals were apparent.

In general, the ratio of CD4^+^/CD8^+^ T-cells is an important diagnostic marker for the function of the immune system, but the ratio varies between the strains. The background strain LEW.1AR1 had a CD4^+^/CD8^+^ ratio of 2∶1 while the ratio in BBDR (diabetes resistant) rats is 3∶1 [34].

Several studies have reported an increased CD4^+^/CD8^+^ ratio also at the onset of human T1DM, either due to a decrease in CD8^+^ T-cells or an increase in CD4^+^ T-cells [35]–[38]. However, during the prediabetic period studies found a normal or decreased CD4^+^/CD8^+^ ratio [39], [40].

In humans an inverse CD4^+^/CD8^+^ ratio is a sign for an acute (auto)immune response. Patients with T1DM showed a lower CD4^+^/CD8^+^ ratio than healthy controls [27], [41]. This finding is in agreement with our results in the LEW.1AR1-*iddm* rat. The low CD4^+^/CD8^+^ ratio may reflect a disturbed immune balance that is primarily due to a decrease in the CD4^+^ T-cells during development of T1DM. The underlying mechanism for this imbalance is still unknown. It is, however, pivotal to know whether the variable T-cell frequency (and the concomitant change of the CD4^+^/CD8^+^ ratio) are genetically inherited and linked to diabetes susceptibility.

### Inheritance of the Variable CD3^+^ T-cell Frequency

The analysis of the CD3^+^ T-cell frequency of the two different backcross populations with the BN and PAR rat strains showed that the variable CD3^+^ T-cell frequency is hereditary. In the N2 generation we found the same variability as in the LEW.1AR1-*iddm* rats that led to a decrease in the CD4^+^/CD8^+^ ratio.

In the human situation the CD4^+^/CD8^+^ ratio is apparently determined by the genetic background as demonstrated in a study on adolescent twins [42]. Other studies have shown that the respective changes in T-cell frequency are also found in non-diabetic relatives [29], [43]. In addition studies in mice and men indicate that the T-cell trait appears to be under the control of a major gene [44], [45]. Interestingly, one major trait locus for the CD4^+^/CD8^+^ ratio in humans is located on chromosome 11p [42]. This region is homologous to the *Iddm8* region on RNO1. Additionally, Ferreira *et al.* showed that there are two QTLs for the CD4^+^/CD8^+^ ratio in humans [46]. One is located in MHC I and affects the CD8^+^ T-cell frequency, while the second is located within the MHC II region, which causes abnormalities in the CD4^+^ T-cell frequency.

### Mapping of the Variable CD3^+^ T-cell Frequency Corresponds to *Iddm8*

Through linkage analyses using microsatellite markers it was possible to identify the *Iddm8* locus within the telomeric end of RNO1 as a susceptibility region not only within the BN but also in the PAR backcross population [7], [10]. Our data clearly favour the *Iddm8* locus as the disease inducing candidate locus causing not only autoimmune diabetes but also the highly variable CD3^+^ T-cell frequency with a decrease of the average T-cell number in diabetic and non-diabetic LEW.1AR1-*iddm* rats.

In the BB rat a mutation of the *Ian5* locus is responsible for the severe loss of peripheral T-cells associated with the diabetes manifestation [47]. In the KDP rat a mutation in the *Cblb* gene affects T-cell activation, thereby triggering T1DM manifestation in this model [47]–[49]. Notably, both these genes were unaffected in the LEW.1AR1-*iddm* rat (data not shown). On the other hand our data clearly indicate that the trait of ‘variable CD3^+^ T-cell frequency’ maps within the *Iddm8* region. This region likely codes for interesting diabetes susceptibility genes, which may have an impact on the T-cell development and function. The study does not allow a differentiation of the CD3^+^ T-cell frequency over the lifespan but there is clear evidence that a genetically-determined variability of the peripheral CD3^+^ T-cells can potentially cause an imbalance between regulatory and effector T-cells.

In summary, the spontaneous mutation in the LEW.1AR1-*iddm* rat leads not only to diabetes manifestation, but also to a highly variable CD3^+^ T-cell frequency and a decrease of the average T-cell number. The mapping of the *Iddm8* region opens the perspective to identify candidate genes for both T-cell variability and diabetes susceptibility by elucidating their interactions in triggering autoimmunity.
